# Antibiotic resistance of *Streptococcus pneumoniae* in Vietnamese children with severe pneumonia: a cross-sectional study

**DOI:** 10.3389/fpubh.2023.1110903

**Published:** 2023-06-13

**Authors:** Khai Tran-Quang, Thuy Nguyen-Thi-Dieu, Hung Tran-Do, Van Pham-Hung, Trung Nguyen-Vu, Bach Tran-Xuan, Mattias Larsson, Sy Duong-Quy

**Affiliations:** ^1^Department of Paediatrics, Can Tho University of Medicine and Pharmacy, Can Tho, Vietnam; ^2^Department of Paediatrics, Hanoi Medical University, Hanoi, Vietnam; ^3^Department of Nursing and Medical Technology, Can Tho University of Medicine and Pharmacy, Can Tho, Vietnam; ^4^International Research of Gene and Immunology Institute, Laboratory of Nam Khoa Biotek Company, Ho Chi Minh City, Vietnam; ^5^Department of Microbiology, Hanoi Medical University, Hanoi, Vietnam; ^6^Department of Health Economics, Institute of Health Economics and Technology, Hanoi Medical University, Hanoi, Vietnam; ^7^Global Public Health Department, Karolinska Institutet, Stockholm, Sweden; ^8^Biomedical Research Center, Lam Dong Medical College, Dalat, Vietnam; ^9^Division of Immuno-Allergology and Pulmonology, Penn State Medical College, Hershey Medical Center, Hershey, PA, United States

**Keywords:** *Streptococcus pneumoniae*, antibiotics resistance, community-acquired pneumonia, children, Vietnam

## Abstract

**Background:**

*Streptococcus pneumoniae* is the most common bacterium that causes community-acquired pneumonia (CAP) in children. The rate of *S. pneumoniae* resistance to antibiotics is increasing, particularly in patients with severe CAP. Therefore, the level of antibiotic resistance of *S. pneumoniae* causing severe CAP in Vietnamese children requires regular monitoring.

**Methods:**

This was a cross-sectional descriptive study. Nasopharyngeal aspiration specimens from children were cultured, isolated, and examined for *S. pneumoniae*. Bacterial strains were assessed for antimicrobial susceptibility, and the minimum inhibitory concentration (MIC) was determined.

**Results:**

Eighty-nine strains of *S. pneumoniae* were isolated from 239 children with severe CAP. The majority of isolates were completely non-susceptible to penicillin (1.1% intermediate, 98.9% resistant) and highly resistant to erythromycin (96.6%) and clarithromycin (88.8%); the rate of resistance to ceftriaxone was 16.9%, with the proportion of intermediate resistance at 46.0%; 100% of strains were susceptible to vancomycin and linezolid. For most antibiotics, MIC_50_ and MIC_90_ were equal to the resistance threshold according to the Clinical and Laboratory Standards Institute 2021; penicillin had an eight-fold increase in MIC_90_ (64 mg/L) and ceftriaxone had a 1.5-fold increase in MIC_90_ (6 mg/L).

**Conclusion:**

*Streptococcus pneumoniae* isolates described in this study were resistant to many antibiotics. Penicillin should not be the first-line antibiotic of choice, and ceftriaxone at an enhanced dose should be used instead.

## Introduction

*Streptococcus pneumoniae* is the most common bacterium that causes community-acquired pneumonia (CAP) in children (1–3). The burden caused by *S. pneumoniae* is strongly related to high morbidity and mortality in children under 5 years of age (4). According to the Global Burden of Disease, Injuries, and Risk Factors Study (GBD), in 195 countries in 2016, *S. pneumoniae* was the cause of more than 341,000 deaths in children under 5 years with lower respiratory tract infections (5).

β-lactam antibiotics are recommended as the primary treatment of infections caused by *S. pneumoniae*. However, since penicillin-non-susceptible pneumococcus was first described in Australia in 1967 (6), the prevalence rate of resistance to β-lactams among *S. pneumoniae* strains has been increasing and has become one of the most important antimicrobial-resistant threats worldwide. Recent reports have shown that the rate of penicillin-non-susceptible *pneumococci* in some countries is 46%, 67.4%, or even up to 97.8% (7–9). Similarly, the emergence of multi-drug resistant (MDR) strains of *S. pneumoniae* has made it difficult to treat diseases caused by this organism (10, 11). Most cases of severe pneumonia fail with initial antibiotic treatment. Data on the role of disease and antibiotic resistance of *S. pneumoniae* causing pneumonia in Can Tho, a major city in southern Vietnam, is outdated and needs updating. This study aimed to evaluate the role of pathogens and the level of antibiotic resistance by determining the minimum inhibitory concentration (MIC) for *S. pneumoniae* causing severe CAP in children in Can Tho City and to clarify the appropriate antibiotic treatment decisions.

## Materials and methods

### Subjects

The study was performed on 239 children with severe CAP admitted to the Department of Respiratory Medicine, General Internal Medicine, Emergency, and Intensive Care Unit of Can Tho Children's Hospital from March 2020 to February 2021. This study was approved by the Institutional Review Board (IRB) of the Ethics Committee of Biomedical Research of Hanoi Medical University, Hanoi, Vietnam (No. 89/GCN-HDDDNCYSH-DHYHN).

### Inclusion criteria

Children aged 2 months to 15 years who were diagnosed with severe pneumonia were included in the present study. The diagnostic criteria of severe CAP were the presence of a cough, dyspnoea and tachypnoea, or chest indrawing, with one of the symptoms such as fever (>38.5°C), hypoxia, cyanosis, short period of breathing pause, dehydration, or oxygen desaturation (< 92%); pneumonia was confirmed by standardized chest X-ray; all the study patients were hospitalized within 48 h.

### Exclusion criteria

Patients who met one of the following criteria were excluded from the study: patients or their parents refused to be involved in the study, patients who had a hospitalized stay during the 14 previous days, and pneumonia due to a non-infectious cause.

### Methods

This was a cross-sectional descriptive study. All patients who met the inclusion criteria were enrolled in the study. The patient underwent clinical examination, biological tests, and chest radiography.

#### The nasotracheal aspiration specimen

Within 48 h, the nasotracheal aspiration (NTA) specimen from each patient was sent to the laboratory for culture and antimicrobial susceptibility testing (12). The quality of the specimens was checked before processing the culture to confirm that the samples originated from the lower respiratory tract. The number of squamous epithelial cells (SECs) and polymorphonuclear cells (PMNs) in the Gram stain smear was counted for each specimen. The presence of < 10 SECs and >25 PMNs per low-power field (magnification, × 100) was considered a high-quality specimen (13).

#### Culture medium

**Figure 1:**
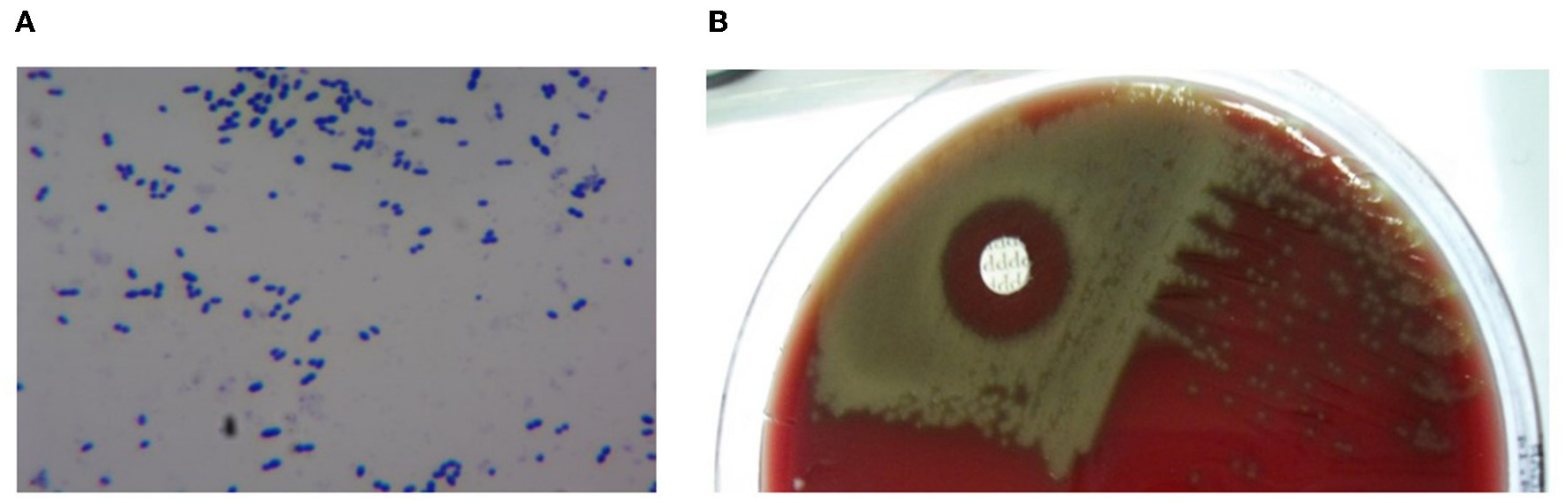
Results of culture of *Streptococcus pneumoniae*. **(A)** Gram-staining; **(B)** Optochin (+).

NTA specimens were cultured on Mueller Hilton blood agar (MHBA) with 5% sheep blood and incubated for 20–24 h at 35°C with 5% CO_2_. Suspected colonies were identified by α-haemolysis, Gram (+) staining, optochin sensitivity, and bile solubility tests (Figure 1) (14).

#### Antimicrobial susceptibility testing

Antimicrobial susceptibility testing (AST) was determined by disc diffusion according to the Kirby-Bauer method (Oxoid Ltd., Basingstoke, UK) for trimethoprim/sulfamethoxazole, chloramphenicol, clindamycin, erythromycin, and oxacillin, and the minimum inhibitory concentration (MIC) was defined as the diffusion of antibiotics in agar from E-test (Bio-Mérieux, Marcy l'Etoile, France) for penicillin, ceftriaxone, ciprofloxacin, levofloxacin, vancomycin, clarithromycin, and linezolid. MIC breakpoints or sterile ring diameters were used to determine antibiotic sensitivity or resistance, as recommended by the manufacturer's instructions for the *E*-test and disc diffusion, as well as by the Clinical and Laboratory Standards Institute (CLSI) in 2021 (14). All isolated organisms were defined as MDR when resistant to ≥3 antibiotic classes (15). MIC_50_ and MIC_90_ were defined as the concentrations at which 50 and 90% of the bacterial strains were inhibited, respectively (14).

### Statistical analyses

Qualitative variables were presented as frequencies and percentages (%). All quantitative variables were presented as mean ± standard deviation or median (min–max). A chi-square test with a 95% confidence interval was used to compare the differences between groups. The *p*-value of < 0.05 was considered significantly different.

## Results

### Clinical and paraclinical characteristics

**Figure 2:**
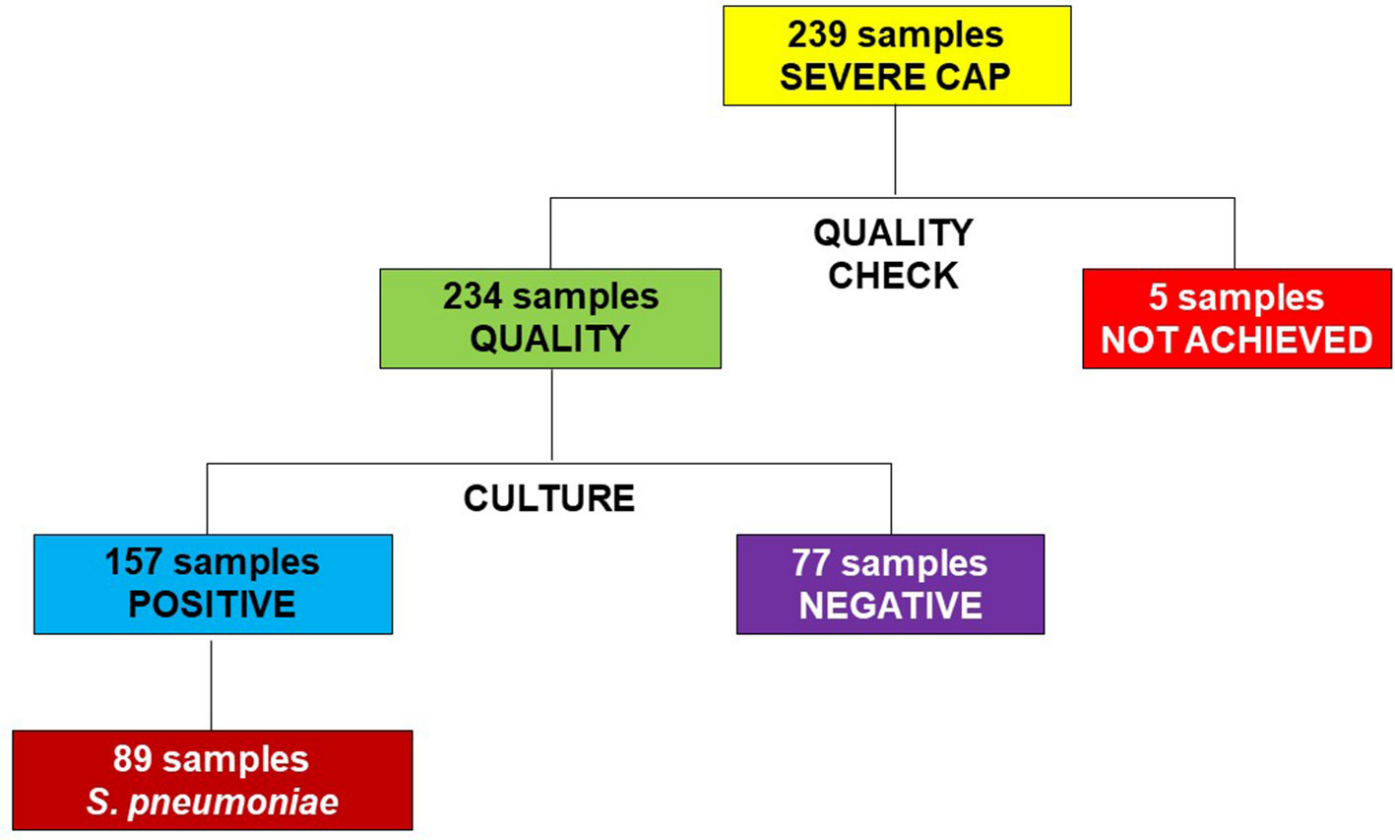
Flowchart of study subjects.

Nasopharyngeal aspiration specimens were collected from 239 children with severe CAP who had been admitted to Can Tho Children's Hospital. Five samples did not meet the required sample quality with >10 squamous cells and < 25 leukocytes, so the culture was not performed; the remaining 234 samples met the required standard. The rate of bacterial isolation was 157/234 (67.1%), of which the percentage of isolated *S. pneumoniae* was 89/234 (38.0%; Figure 2).

The median age of the children with severe CAP caused by *S. pneumoniae* was 17 months; the youngest was 2 months and the oldest was 176 months. The most common age group was under 2 years (64.0%) and rarely occurred in children over 5 years (2.3%). Although severe CAP was more frequent among boys than girls, overall and in episodes caused by *S. pneumoniae*, the percentage of male patients in the complete patients series was significantly higher than in those with severe *S. pneumoniae* CAP (64% vs. 55.1%; *p* = 0.032).

Fever and cough were the two most common symptoms of severe CAP. Most children had physical symptoms in the lungs (90.0% crackles), tachypnoea (88.8%), and chest indrawing (55.1%). Wheezing was less common in children infected with *S. pneumoniae* than in those infected by other microorganisms (*p* = 0.037), and 80.9% of the CAP cases caused by *S. pneumoniae* had peripheral blood oxygen saturation (SpO_2_) ≤ 96%. The mean WBC count was 14.07 ± 5.94 ( × 10^3^/mm^3^). WBC increased by >15.000/mm^3^, accounting for 46.1%; the difference was not statistically significant (*p* = 0.309). Most (>60%) severe CAP cases showed an increase in CRP of >10 mg/L (Table 1).

**Table 1:** Demographic, clinical, and paraclinical characteristics.

**Characteristics**		**Severe CAP caused by *Streptococcus pneumoniae* (*n* = 89) *n* (%)**	**Severe CAP (*n* = 239) *n* (%)**	***p*-value**
Age	< 2 years	57 (64.0)	150 (62.8)	0.223
	2–5 years	30 (33.7)	76 (31.8)	
	>5 years	2 (2.3)	13 (5.4)	
Sex	Male	49 (55.1)	153 (64)	**0.032**
	Female	40 (44.9)	86 (16)	
Pneumococcal vaccination	Complete	3 (3.4)	3 (1.3)	0.412
	Not available	81 (91.0)	223 (93.3)	
	Not clear	5 (5.6)	14 (5.9)	
Symptoms and signs	Fever	89 (100)	239 (100)	NA
	Cough	89 (100)	239 (100)	NA
	Vomiting	11 (12.4)	29 (12.1)	0.909
	Diarrhea	8 (9.0)	24 (10.0)	0.507
	Chest pain	3 (3.4)	11 (4.6)	0.571
	Abdominal pain	2 (2.3)	3 (1.3)	0.904
	Tachypnoea	79 (88.8)	214 (89.5)	0.833
	Chest indrawing	49 (55.1)	136 (56.9)	0.794
	Accessory muscle used	38 (42.7)	88 (36.8)	0.223
	Crackles	80 (90.0)	212 (88.7)	0.429
	Wheezing	29 (32.6)	141 (59.0)	**0.037**
	SpO_2_ ≤ 96%	72 (80.9)	194 (81.2)	0.701
WBC count	>15,000/mm^3^	41 (46.1)	99 (41.4)	0.309
CRP	>10 mg/L	58 (65.2)	144 (60.3)	0.169

WBC, white blood cell; CRP, C-reactive protein; NA, not applicable. The bold values mean statistically significant difference (with *p* < 0.05).

### Antibiotic-resistant characteristics

All isolates of *S. pneumoniae* were identified as MDR strains. These strains were completely non-susceptible to penicillin; for ceftriaxone, the majority of strains were intermediate (46.0%), followed by susceptible (37.1%); 100% of the strains were susceptible to vancomycin and linezolid (Table 2).

**Table 2:** Antibiotic susceptibility patterns of *Streptococcus pneumoniae* isolates from children (*n* = 89).

**Antibiotics**	**Susceptibility**			***p*-value**
	**S *n* (%)**	**I *n* (%)**	**R *n* (%)**	
Trimethoprim/ sulfamethoxazole	7 (7.9)	2 (2.2)	**80 (89.9)**	0.001
Clindamycin	10 (11.2)	0	**79 (88.8)**	0.001
Erythromycin	1 (1.1)	2 (2.2)	**86 (96.6)**	0.001
Clarithromycin	5 (5.6)	5 (5.6)	**79 (88.8)**	0.001
Penicillin	0	1 (1.1)	**88 (98.9)**	0.001
Ceftriaxone	**33 (37.1)**	**41 (46.0)**	**15 (16.9)**	0.001
Chloramphenicol	**84 (94.4)**	0	5 (5.6)	0.001
Ciprofloxacin	**53 (59.6)**	6 (6.7)	30 (33.7)	0.001
Levofloxacin	**72 (80.9)**	4 (4.5)	13 (14.6)	0.001
Vancomycin	**89 (100)**	0	0	0.001
Linezolid	**89 (100)**	0	0	0.001

S, susceptible; I, intermediate; R, resistant. The bold values mean statistically significant difference (with *p* < 0.05).

Ceftriaxone-non-susceptible strains were more resistant to ciprofloxacin than ceftriaxone-susceptible strains, and this difference was statistically significant (*p* = 0.048; Table 3).

**Table 3:** Susceptibility patterns of ceftriaxone-susceptible *Streptococcus pneumoniae* (*n* = 33) and ceftriaxone-non-susceptible *S. pneumoniae* (*n* = 56).

**Antibiotics**	**Ceftriaxone-susceptible** ***S. pneumoniae*** **(*****n*** = **33)**			**Ceftriaxone-non-susceptible** ***S. pneumoniae*** **(*****n*** = **56)**			***p*-value**
	**S *n* (%)**	**I *n* (%)**	**R *n* (%)**	**S *n* (%)**	**I *n* (%)**	**R *n* (%)**	
Trimethoprim/ sulfamethoxazole	4 (12.1)	1 (3.0)	28 (84.9)	3 (5.4)	1 (1.8)	52 (92.8)	0.473
Clindamycin	2 (6.1)	0	31 (93.9)	8 (14.3)	0	48 (85.7)	0.235
Erythromycin	0	0	33 (100)	1 (1.8)	2 (3.6)	53 (94.6)	0.401
Clarithromycin	0	1 (3.0)	32 (97.0)	5 (8.9)	4 (7.2)	47 (83.9)	0.137
Chloramphenicol	31 (93.9)	0	2 (6.1)	53 (94.6)	0	3 (5.4)	0.889
Ciprofloxacin	**25 (75.8)**	**1 (3.0)**	**7 (21.2)**	**28 (50.0)**	**5 (8.9)**	**23 (41.1)**	**0.048**
Levofloxacin	26 (78.8)	2 (6.1)	5 (15.1)	46 (82.1)	2 (3.6)	8 (14.3)	0.850
Vancomycin	41 (100)	0	0	48 (100)	0	0	NA
Linezolid	41 (100)	0	0	48 (100)	0	0	NA

S, susceptible; I, intermediate; R, resistant; NA, not applicable. The bold values mean statistically significant difference (with *p* < 0.05).

### Minimal inhibitory concentration (MIC) distribution

Table 4 shows the MIC distribution of the antibiotics, and Table 5 shows the MIC_50_ and MIC_90_ values of the antibiotic tested against *S. pneumoniae*. For most antibiotics, MIC_50_ coincided with MIC_90_ and equaled the resistance threshold indicated in CLSI 2021; penicillin had an eight-fold increase in MIC_90_ (64 mg/L) and ceftriaxone had a 1.5-fold increase in MIC_90_ (6 mg/L).

**Table 4:** MIC distribution of *Streptococcus pneumoniae* isolates from children (*n* = 89).

**Antibiotics**	**Number of isolates at MIC values (mg/L)**
	**0.18**	**0.25**	**0.38**	**0.5**	**0.75**	**1**	**1.5**	**2**	**3**	**4**	**6**	**8**	**12**	**16**	**24**	**32**	**48**	**64**	**128**
Trimethoprim/ sulfamethoxazole^*^				7		2				**80**									
Clindamycin				10		**79**													
Erythromycin		1		2		**86**													
Clarithromycin		5		5		**79**													
Penicillin										1	1	3	1	1	1	3	4	**74**	
Ceftriaxone	4	1	4	4	5	15	9	**27**	5	4	6	5							
Chloramphenicol										**84**		5							
Ciprofloxacin	4		2	10	9	2	**24**	10	1	9	4	4	4	2	1	1	1		1
Levofloxacin	3	5	15	**19**	14	2	6	9	2	3	2	2	4	2	1				
Vancomycin				1		**88**													
Linezolid								**89**											

MIC, minimal inhibitory concentration.

^*^MIC values were shown in the table for Trimethoprim, corresponding to the MIC of Trimethoprim/sulfamethoxazole 0.5/9.5, 1/19, 2/38, 4/76 (mg/L). The bold values are the frequencies for the values of MIC90, the concentration at which 90% of bacterial strains are inhibited.

**Table 5:** MIC breakpoints according to CLSI in 2021 and MIC_50_; MIC_90_ of antibiotics for isolated *Streptococcus pneumoniae*.

**Antibiotics**	**MIC breakpoints according to CLSI in 2021 (mg/L)**			**MIC_50_ (mg/L)**	**MIC_90_ (mg/L)**
	**S**	**I**	**R**		
Trimethoprim/sulfamethoxazole	≤ 0.5/9.5	1/19–2/38	≥4/76	4/76	4/76
Clindamycin	≤ 0.25	0,5	≥1	1	1
Erythromycin	≤ 0.25	0,5	≥1	1	1
Clarithromycin	≤ 0.25	0,5	≥1	1	1
Penicillin (non-meningitis)	≤ 2	4	≥8	64	64
Ceftriaxone (non-meningitis)	≤ 1	2	≥4	2	6
Chloramphenicol	≤ 4	–	≥8	4	8
Ciprofloxacin	NT	NT	NT	1.5	12
Levofloxacin	≤ 2	4	≥8	0.75	8
Vancomycin	≤ 1	–	**–**	1	1
Linezolid	≤ 2	–	–	2	2

MIC, Minimal Inhibitory Concentration; MIC_50_, concentration at which 50% of bacterial strains were inhibited; MIC_90_, concentration at which 90% of bacterial strains were inhibited; CLSI, clinical and laboratory standards institute; S, susceptible; I, intermediate; R, resistant; NT, not tested.

## Discussion

Bacterial culture is the ‘gold standard' for the identification of pneumonia pathogens (17). In this study, the rate of isolation of bacterial agents of pneumonia was 67.1%, which is in agreement with others (18) and higher than others, with rates of 35% and 42% (19, 20). However, the rate reported here was lower than the one reported by Olwagen et al. (21) of 71%. Obviously, the rate of positive bacterial cultures varies widely by studies and countries and is dependent on sampling techniques, storage, culture, incubation, and temperature used, for which standardized techniques are required when working with such specimens (17). In addition, the present study was conducted with patients with severe pneumonia, which explains why the rate of isolated bacteria was so high.

The present study showed that severe CAP caused by *S. pneumoniae* (and other microorganisms) was common, mainly in children under 2 years of age, and followed by 2–5 years. This finding is consistent with what is in literature, with many studies recognizing that children under 5 years of age are more susceptible to pneumonia than older children, and tend to suffer a worse prognosis of the disease (18, 22). The proportion of boys with severe CAP due to *pneumococci* was higher than in girls, a finding which is in agreement with other studies (23, 24). Of the children with severe CAP, 93.3% did not have pneumococcal immunization status and only three (1.3%) were vaccinated. However, all these three were infected with *pneumococci*, which begs the question of which *S. pneumoniae* serotypes infected the children in the study group and that matched the serotypes of the vaccine used? This is also a limitation of this study, which is being implemented as a follow-up study in this area. A study in a northern province of Vietnam identified eight serotypes of *S. pneumoniae* isolated from unvaccinated children under 5 years of age with pneumonia, including 19F, 23F, 19A, 6A/B, 15A, 9V, 11A, and 14 (25).

The present study showed that most children with CAP caused by *S. pneumoniae* had peripheral blood oxygen saturation (SpO_2_) ≤ 96%. Some recent studies have shown that there are no signs or symptoms sufficient to confirm pneumonia in children, but diagnostic specificity can be improved when some clinical features, such as tachypnoea, fever, and hypoxia, are present (26, 27). A decline in SpO_2_ is also a valuable parameter for diagnosing pneumonia. According to Shah et al. (28), the presence of moderate hypoxaemia and increased breathing effort are the most relevant signs of pneumonia. In the present study, wheezing was less common in children infected with *S. pneumoniae* than in those infected by other microorganisms. A previous study demonstrated that wheezing was more common in atypical or viral pneumonia than in typical bacterial pneumonia (29).

The present study suggests that WBC is not a reliable indicator of the severity of the disease (Table 1), which is consistent with a previous study showing that WBC was a poor indicator of the etiology and severity of pneumonia (24). Esposito et al. (30) highlighted that WBC count had the lowest positive predictive value compared to procalcitonin and CRP. The present study found that up to 65.2% of children with severe CAP due to *S. pneumoniae* had an increase in CRP levels >10 mg/L. CRP is also considered an acute-phase reactant associated with disease severity in children with bacterial infection (31).

In this study, the majority of *S. pneumoniae* isolates were identified as MDR, with 98.9% of the isolates completely non-susceptible to penicillin and others. In recent years, the proportion of penicillin non-susceptible *pneumococci* has increased to over 60% (7–9). Consequently, penicillin is no longer the first-line antibiotic for children with severe CAP in many countries; clinicians are using cefotaxime and ceftriaxone, a third-generation cephalosporin, instead (32, 33). In this study, the rate of *S. pneumoniae* resistant to ceftriaxone was only 16.9%; however, the percentage of intermediate resistance was 46.0%. This result raises great concern about the likelihood of first-line antibiotic failure. When comparing the sensitivity of antibiotics between the two groups of ceftriaxone-susceptible and ceftriaxone-non-susceptible *S. pneumoniae*, ceftriaxone-non-susceptible strains were still sensitive to vancomycin (100%), linezolid (100%), and chloramphenicol (93.8%). A previous study on antimicrobial susceptibility testing for isolated *S. pneumoniae* also showed a low susceptibility rate to all antibiotics except vancomycin (100%), linezolid (100%), and levofloxacin (89.5%) (34). According to the results of a study conducted by Sweden et al., the proportion of MDR *S. pneumoniae* susceptible to vancomycin and levofloxacin was 100% (16). However, chloramphenicol is rarely used orally and intravenously in Vietnamese children due to its common side effects such as blood dyscrasias, aplastic anemia, and leukemia (35, 36). Therefore, vancomycin and linezoild may be suitable alternative antibiotics for severe MDR pneumococcal pneumonia.

Macrolide antibiotics have become less effective against *S. pneumoniae*, with high resistance rates reported in the present study for erythromycin (96.6%) and clarithromycin (88.8%). Other studies have found that *S. pneumoniae* is more resistant to macrolides and does not support the routine combination of β-lactam antibiotics with macrolides (16, 37). However, in contrast, other studies have suggested that macrolide combination therapy is more effective than β-lactam monotherapy, especially in children >5 years of age or in atypical bacterial co-infections with pathogens such as *Mycoplasma pneumoniae* (38, 39).

Analysis of the distribution of MIC, MIC_50_, and MIC_90_ among the studied *S. pneumoniae* isolates revealed that penicillin had an eight-fold increase in MIC_90_ (64 mg/L). This finding suggests that ceftriaxone is more suitable than penicillin for the initial treatment of severe CAP caused by *S. pneumoniae*. However, based on our study findings, an increase in the dose of ceftriaxone is needed, as evidenced by the 1.5-fold increase in MIC_90_ for this antibiotic in this study. Recommendations for how much to increase the dose depends on the pharmacokinetic/pharmacodynamic (pK/pD) breakpoint, which is another interesting follow-up to this study. In recent years, many recommendations have been made to increase the therapeutic dose of ceftriaxone to 100 mg/kg/day for severe infections or in places with insignificant penicillin resistance (≥25%) (33, 40).

In the present study, the fluoroquinolones, ciprofloxacin and levofloxacin, had a fairly wide MIC range of 0.18–128 and 0.18–24 mg/L, respectively. These results suggest that it may be difficult to choose a therapeutic dose of ciprofloxacin. Moreover, if a strain of *S. pneumoniae* was not susceptible to ceftriaxone, it was likely that it would not be susceptible to ciprofloxacin, although the rate of ciprofloxacin susceptibility to *S. pneumoniae* was 59.6%. Although there have been very few reports of treatment failure with ciprofloxacin, this antibiotic was not considered a “respiratory fluoroquinolone” in the United States (41). Indeed, the CLSI does not provide ciprofloxacin breakpoints for *S. pneumoniae* (14). Furthermore, ciprofloxacin is not recommended for the treatment of respiratory tract infections in most treatment guidelines (33, 42). In fact, the systemic use of fluoroquinolones in patients under 18 years is not recommended in many countries due to eventual adverse musculoskeletal effects (43). Therefore, extreme caution should be exercised when using ciprofloxacin to treat children with severe CAP.

## Conclusion

Our study showed a high prevalence rate of *S. pneumoniae* strains resistant to many antibiotics, including penicillin-resistant strains with high MICs, among strains from children with severe CAP. Therefore, penicillin should not be the first-line antibiotic of choice, and ceftriaxone at an enhanced dose should be used instead.

## Data availability statement

The original contributions presented in the study are included in the article/supplementary material, further inquiries can be directed to the corresponding author.

## Ethics statement

The studies involving human participants were reviewed and approved by Review Board Committee for Ethics Committee in Biomedical Research of Hanoi Medical University, Hanoi, Vietnam (No. 89/GCN-HDDDNCYSH-DHYHN). Written informed consent to participate in this study was provided by the participants' legal guardian/next of kin.

## Author contributions

Conceptualization, resources, and investigation: KT-Q, TN-T-D, HT-D, and VP-H. Software: HT-D, BT-X, ML, and SD-Q. Formal analysis: TN-T-D, SD-Q, HT-D, TN-V, BT-X, and ML. Manuscript writing, review, and editing: KT-Q, TN-T-D, HT-D, TN-V, BT-X, ML, and SD-Q. All authors have read, commented upon, and approved the final manuscript.
